# Divergence of a genomic island leads to the evolution of melanization in a halophyte root fungus

**DOI:** 10.1038/s41396-021-01023-8

**Published:** 2021-06-09

**Authors:** Zhilin Yuan, Irina S. Druzhinina, John G. Gibbons, Zhenhui Zhong, Yves Van de Peer, Russell J. Rodriguez, Zhongjian Liu, Xinyu Wang, Huanshen Wei, Qi Wu, Jieyu Wang, Guohui Shi, Feng Cai, Long Peng, Francis M. Martin

**Affiliations:** 1 State Key Laboratory of Tree Genetics and Breeding, Chinese Academy of Forestry, Beijing, China; 2 Research Institute of Subtropical Forestry, Chinese Academy of Forestry, Hangzhou, China; 3 Fungal Genomics Laboratory (FungiG), College of Resources and Environmental Sciences, Nanjing Agricultural University, Nanjing, China; 4 Department of Food Science, University of Massachusetts, Amherst, MA USA; 5 State Key Laboratory of Ecological Pest Control for Fujian and Taiwan Crops, College of Plant Protection, Fujian Agriculture and Forestry University, Fuzhou, China; 6 Department of Molecular, Cell and Developmental Biology, University of California, Los Angeles, CA USA; 7 Department of Plant Biotechnology and Bioinformatics, Ghent University, Ghent, Belgium; 8 VIB Center for Plant Systems Biology, Ghent, Belgium; 9 Centre for Microbial Ecology and Genomics, Department of Biochemistry, Genetics and Microbiology, University of Pretoria, Hatfield, South Africa; 10 Adaptive Symbiotic Technologies, University of Washington, Seattle, WA USA; 11 Key Laboratory of National Forestry and Grassland Administration for Orchid Conservation and Utilization at College of Landscape Architecture, Fujian Agriculture and Forestry University, Fuzhou, China; 12 State Key Laboratory of Mycology, Institute of Microbiology, Chinese Academy of Sciences, Beijing, China; 13 Key Laboratory of Plant Resources Conservation and Sustainable Utilization, South China Botanical Garden, Chinese Academy of Sciences, Guangzhou, China; 14 Beijing Advanced Innovation Center for Tree Breeding by Molecular Design, Beijing Forestry University, Beijing, China; 15 Université de Lorraine, INRAE, UMR Interactions Arbres/Micro-Organismes, Centre INRAE Grand Est Nancy, Champenoux, France

**Keywords:** Population dynamics, Fungal ecology, Population genetics

## Abstract

Understanding how organisms adapt to extreme living conditions is central to evolutionary biology. Dark septate endophytes (DSEs) constitute an important component of the root mycobiome and they are often able to alleviate host abiotic stresses. Here, we investigated the molecular mechanisms underlying the beneficial association between the DSE *Laburnicola rhizohalophila* and its host, the native halophyte *Suaeda salsa*, using population genomics. Based on genome-wide Fst (pairwise fixation index) and Vst analyses, which compared the variance in allele frequencies of single-nucleotide polymorphisms (SNPs) and copy number variants (CNVs), respectively, we found a high level of genetic differentiation between two populations. CNV patterns revealed population-specific expansions and contractions. Interestingly, we identified a ~20 kbp genomic island of high divergence with a strong sign of positive selection. This region contains a melanin-biosynthetic polyketide synthase gene cluster linked to six additional genes likely involved in biosynthesis, membrane trafficking, regulation, and localization of melanin. Differences in growth yield and melanin biosynthesis between the two populations grown under 2% NaCl stress suggested that this genomic island contributes to the observed differences in melanin accumulation. Our findings provide a better understanding of the genetic and evolutionary mechanisms underlying the adaptation to saline conditions of the *L. rhizohalophila–S. salsa* symbiosis.

## Introduction

The process by which organisms become better adapted to extreme environments and how natural selection shaped organismal phenotypes and genotypes in response to these intense selective pressures are central to evolutionary biology. Melanin-based pigmentation has long been used as a trait to understand adaptive evolution in extreme habitats across all forms of life [1–4]. Like other organisms, fungi are known to adapt to many stressful environments [5]. Survival mechanisms often involve a higher melanin biosynthesis [6–8], leading to increased cell wall rigidity, resistance to many types of environmental stresses, including inactivation of the plant defense systems [6]. Genome-based approaches have been used to investigate the evolution and adaptation of melanized fungi, mostly model species of plant and human pathogens, and soil fungi [9–13]. As of today, our understanding of the molecular mechanisms underlying the role of melanin in the adaptation of plant-associated mutualistic fungi to extreme environments is scarce [14–16].

Endophytic fungi often represent a very prominent component of the plant root mycobiome [17] and their diversity and ecological significance have only recently been widely appreciated [18]. In particular, the root DSEs have received increased attention because they are ubiquitous in terrestrial ecosystems [19], and can decay organic matter and promote host stress tolerance [20–22]. Of note, DSEs are highly abundant in extreme environments experiencing low/high temperatures, drought, or/and high salinity. This prominence suggests that they might possess unique, undiscovered functional traits [23]. The main unifying and conspicuous feature of DSEs is their ability to produce melanized hyphae and microsclerotia in the colonized roots of their host(s) [19, 24]. It has thus been suggested that melanization is a key trait for salt tolerance [25]. Therefore, the study of the evolution of melanin biosynthesis pathways in DSEs should provide a better understanding of the molecular mechanisms underlying ecological adaptations.

Prior to this study, we performed a survey of the endophytic fungi colonizing roots of the native halophyte *Suaeda salsa* [26], a widely distributed plant in the saltmarsh areas along the western coast of the Yellow Sea in China. We found that a novel haploid DSE species, *Laburnicola rhizohalophila* sp. nov. (Pleosporales, Ascomycota) was a core member of the root mycobiome [26, 27]. Intriguingly, *L. rhizohalophila* isolates displayed a high degree of within*-*population phenotypic and physiological variations [27]. This finding was unexpected owing to the fact that genotyped individuals were isolated from *S. salsa* seedlings collected less than one km apart. To address the potential influence of salt stress on *L. rhizohalophila* population structure, we confirmed the population structure by genome sequencing. Then, we assessed whether some genomic regions are experiencing positive selection and are responsible for the control of adaptive phenotypic traits. Furthermore, we characterized the functional relevance of such sequence variations to provide experimental evidence for selection-associated traits. Our work serves as a case study for investigating the mechanisms underlying the adaptation to abiotic stresses in a poorly investigated, but ecologically important, guild of plant-associated endophytes. This work also emphasizes the importance of taking a “reverse-ecology” approach linking “a priori” genome scan and “a posteriori” phenotypic characterization [28], albeit this approach is not in wide use.

## Materials and methods

### *L. rhizohalophila* isolates

*S. salsa* is an annual herbaceous euhalophyte that grows in saline soils. The present isolates were sampled along the coastal area in Dongying (N37*°*23′43′′, E118 *°*55′25′′), Shandong province, China. We collected *S. salsa* roots in July 2013 and 2014, and in June 2015. The distance between the three randomly selected sampling sites was less than 1.0 km. All endophytic *L. rhizohalophila* strains were isolated from surface-sterilized root tissues. The detailed isolation protocol was previously described [26]. In total, 29 isolates were used for the present population genomics analysis. Ex-type living cultures of *L. rhizohalophila* are preserved in the China General Microbiological Culture Collection Center (CGMCC 3.19607–CGMCC 3.19616 and CGMCC 8756) [27].

### Fungal DNA preparation and genome sequencing

We used *L. rhizohalophila* JP-R-44 as the reference isolate for high-quality de novo genome sequencing because JP-R-44 is the holotype for this novel species [27]. Protocols used for genomic DNA extraction, DNA library construction, genome sequencing, genome assembly, and annotation are provided in the Supporting Information text S1.

### Whole-genome and whole-population Illumina sequencing

The nuclear genomes of 29 *L. rhizohalophila* isolates were subjected to Illumina sequencing. A library with insert sizes of 300 bp was constructed using the NEBNext Ultra II DNA Library Prep Kit (New England BioLabs, Massachusetts, USA). The libraries were then sequenced on a HiSeq X Ten System (Illumina) using a PE-150 module.

### SNP calling and population genetic analyses

For SNP calling, filtered high-quality DNA sequencing reads of the 29 individuals were aligned to the JP-R-44 reference genome using BWA software v0.7.17 and the MEM module [29]. SAM alignment files were sorted and converted to BAM files with SAMtools v1.3 [30]. Next, HaplotypeCaller, CombineGVCFs, GenotypeGVCFs, SelectVariants, and VariantFiltration in the GATK package were used to call, filter, and select SNPs [31]. The filter parameters were as follows: “QD < 2.0 | | FS > 80.0 | | MQ < 20.0 | | SOR > 3.0 | | MQRankSum < −12.5 | | ReadPosRankSum < −8.0 | | QUAL < 40.0”.

To infer the phylogenomic relationships among individuals, a neighbor-joining phylogenetic tree was constructed by TreeBest v1.9.2. (http://treesoft.sourceforge.net/treebest.shtml) under the *p*-distances model using population-scale SNPs. The population structure was first studied using principal component analysis (PCA). We performed PCA with population-scale SNPs using the software *plink* [32] and *gcta* [33]. A Bayesian population structure assessment was inferred using ADMIXTURE v1.3.0 [34] with maximum likelihood estimation and the block relaxation algorithm. Simulations were run with 1000 bootstraps and tenfold cross-validation. We increased the pre-defined genetic clusters from *K* = 2 to *K* = 10 (number of ancestral populations). Prior to running ADMIXTURE, we removed SNPs that were in high linkage disequilibrium (LD). Specifically, we used *plink* to prune SNPs using a windowed approach, along with an *R*^2^ threshold of 0.8.

### Analysis of LD decay and recombination detection

To determine whether the population structure of *L. rhizohalophila* was clonal, panmictic, or selfing, we performed an LD decay analysis. Pairwise LD was calculated between all SNPs from 1 and 10,000 bp apart using *vcftools* with the settings “vcf All.recode.vcf–keep cleda1.lst–ld-window-bp 10,000–min-r2 0–geno-r2–out cleda1.10k”. The mean value of LD across 10-kbp sliding windows from any given SNP was then calculated. Loess curves were fitted to the mean LD decay values for the sliding windows with the *R* function “loess”. To determine the approximate distance required to reach 50% LD decay, we used the window start of the closest point above 50% of the maximum linkage. In the scenario that one of the populations had a significantly higher number of SNPs than the other, we would have a finer resolution to detect recombination events. To address this scenario, we used the identical number of SNPs (170,000) randomly sampled along the genome from group 1 and group 2 for the LD analysis.

To determine the level of recombination between groups, we visualized potential recombination events in SplitsTree v4.14.4 (http://splitstree.org/) [35] for the whole dataset using pairwise distances with the Kimura K3ST model, which allows for the identification of reticulated evolution rather than a strictly bifurcating path.

### Identification of the mating-type (*MAT*) locus and flanking regions in *L. rhizohalophila* populations

The sequence of the *MAT* locus and its flanking regions in the JP-R-44 genome was determined by performing a local tBlastn search [36]. The identities of the annotated genes were further determined by BLASTp against the National Center for Biotechnology Information (NCBI) non-redundant protein database [37].

### Estimation of population differentiation

We used three indices to assess population differentiation: SNP analysis using Fst (Wright’s fixation index, mean genetic differentiation) [38] and Dxy (absolute genetic divergence) (equation 10.20) [39], and copy number variation (CNV) analysis using Vst (a population differentiation estimator similar to Fst). We selected overlapping 10-kbp windows with a 1-kbp step size to identify regions with increased genetic divergence (Fst) across the two groups as indicated by the ADMIXTURE analysis. This window size was chosen to provide a reasonably large number of SNPs per window (average 108 SNPs per window in *L. rhizohalophila* population) as recommended in [40]. The *gl.fst.pop* script was used to calculate pairwise Fst values in the R package *StAMPP* with 1000 bootstrap replications [41]. Dxy was calculated with an in-house script. We Z-transformed the distribution of Fst and the ZFst values were standardized using R [42]. Genomic windows with high ZFst values (the top 1% and 5%) were used as the significance levels and analyzed for gene content. Candidate loci were visualized using Manhattan plots. Gene ontology (GO) term enrichment analysis in the list of differentiated genes was performed with clusterProfiler v3.4.4 [43]. We set the significant threshold of GO terms enrichment to *p* value ≤ 0.05.

To identify whether CNVs were also divergent between the two populations, we first identified windows showing significant variation in normalized read depth. Here, we refer to CNV of 0 as an “absence” rather than a deletion because the absence of a locus in an analyzed genome could be the result of a gain in the reference genome and not solely a deletion in the analyzed genomes [44]. To screen genomes for CNVs, we used the *cn.MOPS* R package with the *haplocn.mops* algorithm (the default bin size was 1 kbp), which is specifically designed for haploid organisms [45]. We plotted CNV heatmaps for the entire genome as well as heatmaps for genes of interest. In addition, we calculated two parameters, including the polymorphic index content (PIC) and Vst, to measure CNV diversity and divergent CNV profiles between the groups, respectively [44].

### Detection of genomic signatures of selection

To identify the potential evolutionary processes affecting population differentiation, we used population-based methods to detect selection on coding sequences. We tested for departures from neutrality using Tajima’s D statistics [46] to infer neutrality and/or selection at genomic regions with ZFst outliers, implying divergent natural selection. Tajima’s D detects departures from the expected site-frequency spectrum under neutral assumptions by comparing two measures of nucleotide genetic diversity (*θ*) [47]—segregating sites (*θ*_*W*_) and nucleotide diversity (*θπ*)—which were calculated using VariScan v2.0.2 software [48]. As Fst statistics are sometimes affected by demography, migration, and small sample size [49], we applied a nonparametric empirical permutation approach to obtain a null distribution of Tajima’s D statistics [50]. Then, we compared this distribution to the observed Tajima’s D statistics to identify candidate regions under selection for each group. Briefly, when the statistical values of a given locus were below the fifth percentile of the genomic and null distribution, we concluded that the signal of positive selection was active and strong [51, 52]. To further confirm the positive selection signals identified, we used the composite likelihood ratio (CLR) test [53] as implemented in the program SweeD v3.3.2 [54]. An overlapping 10-kbp window across the whole genome was used for scanning with a cross-population composite likelihood (XP-CLR) test. Finally, selective sweeps often leave large areas surrounding the site of selection in LD. We used Haploview v4.1 [55] to visualize LD in the candidate genomic regions where extremely negative Tajima’s D values were observed.

### Mycelial growth and melanin biosynthesis under salinity conditions

We used the mycelial growth and melanin biosynthesis under saline conditions as proxies for fitness. Seven isolates from group 1 or group 2 of *L. rhizohalophila* were randomly selected for phenotypic measurements. We used potato dextrose broth (PDB) medium with or without 2% (w/v) NaCl for culturing the vegetative mycelium. All fungal cultures were kept in an incubator without shaking for up to 2 weeks at 25 °C. Each treatment was conducted in four biological replicates. Detailed methods for melanin extraction and measurement have been described in [56].

Biomass accumulation and colony diameter were used to measure fungal growth. Biomass measurements were taken as described above using the same culture conditions, but isolates were grown on PDA with 2% or 6% NaCl. Colony diameters were measured in triplicate after two weeks at 25 °C. The software OriginLab 10.0 (OriginLab Corporation, Northampton, USA) was used for statistical analysis and plotting. Significant differences between the two *L. rhizohalophila* groups were evaluated with a Student’s *t*-test at *p* ≤ 0.05. All data were expressed as mean values with standard deviations (SD).

### Data accessibility

The genome assembly and annotation of *L. rhizohalophila* JP-R-44 are available at the NCBI (BioProject number: PRJNA517533, BioSample number: SAMN10836428). The whole-genome sequencing (WGS) project has been deposited at DDBJ/ENA/GenBank under the accession SELF00000000. The raw genome resequencing data have been deposited at the NCBI in the sequence read archive (SRA) database under the accession numbers: SRR8569110 to SRR8569138. Raw VCF file (SNP variants) is available via Data Dryad (10.5061/dryad.18931zcvw).

## Results

### Genome and population structure of *L. rhizohalophila*

**Fig. 1:**
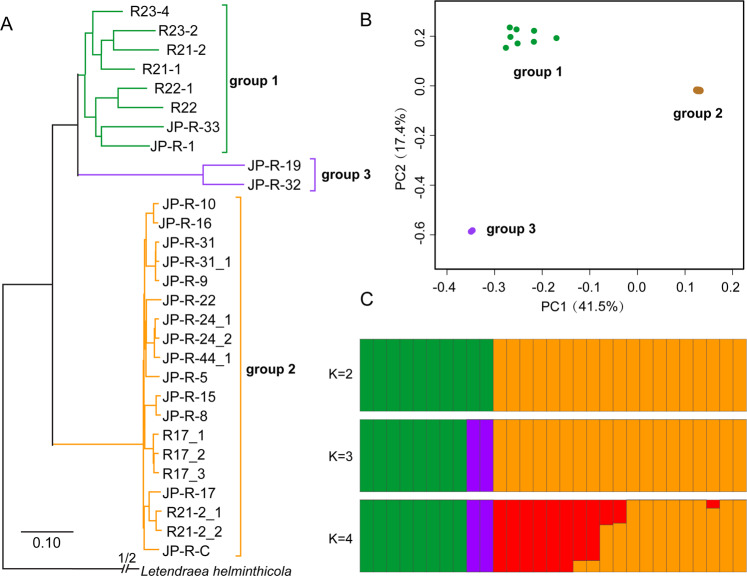
Genetic structure of *Laburnicola rhizohalophila* population. **A** Phylogenomic relationships of *L*. *rhizohalophila* isolates, and the outgroup species *Letendraea helminthicola* (CBS 884.85, Pleosporales, Ascomycota). The maximum likelihood (ML) tree was constructed using 1,241,238 SNPs. The tree groups (or subpopulations) are indicated by red, blue, and purple colors. **B** Principal component analysis (PCA) of 29 individual isolates identified three distinct groups. Individuals within the same population are marked using the same symbols. The first and second principal components account for 41.5% and 17.4% of the variation, respectively. **C** ADMIXTURE plots for increasing numbers of clusters (K is set from 2 to 4). Simulations were set at 1000 bootstraps with tenfold cross-validation. Each individual is represented by a thin vertical bar, which is partitioned into K-colored segments and represents the individual affiliation to each cluster.

The *L. rhizohalophila* reference genome was assembled into 42 scaffolds with a cumulative length of 61.4 Mbp and N50 of 2.53 Mbp. It contains 14,646 protein-coding genes. The quality of the genome assembly and gene annotation were evaluated with BUSCO (Benchmarking Universal Single-Copy Orthologs v4.0.1, Fungi odb10 dataset). The 90.2% completeness suggests that the genome assembly and annotation are of suitable high quality (Table S1). Whole-genome resequencing from 29 individuals produced > 0.9 billion reads with an average sequencing depth of 72× per individual, ranging from 41× to 159× (Table S2). Sequencing reads were aligned to the reference genome for SNP calling. We recovered a total of 1,241,238 SNPs across the 29 *L. rhizohalophila* genomes after singleton filtering, with 2.0% of the genome as variable sites. The distance-based NJ phylogenetic tree revealed that the *L. rhizohalophila* population was well separated into three major subgroups with group 1 genetically closer to group 3, and distant to group 2 (Fig. 1A).

A genetic structure with three subpopulations was further confirmed by using PCA (Fig. 1B) and admixture*-*based model (Figs. 1C and S1) analyses; the first two principal components (axis 1 and axis 2) accounted for ~59% of the cumulative genetic variation (Fig. 1B). Group 3 was excluded from subsequent analysis due to the small sample size (*N* = 2).

### Recombination pattern analysis and MAT gene structure

**Fig. 2:**
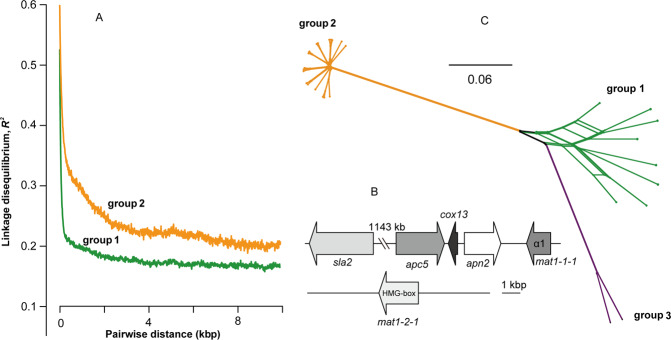
Analysis of the recombination pattern in the *L*. *rhizohalophila* population. **A** Linkage disequilibrium (LD) decay between pairs of SNPs, measured as *R*^2^, with distance on the same scaffold. **B** Mating structure and organization of *L*. *rhizohalophila* JP-R-44 suggested its homothallic reproduction mode. *sla2*: cytoskeleton assembly control protein, (protein ID 13068); *apn2*: DNA lyase (protein ID 13428); *cox13*: cytochrome oxidase (protein ID 13427); *apc5*: amino acid-polyamine-organocation (protein ID 13426). **C** Phylogenetic network generated in SplitsTree of 29 *L*. *rhizohalophila* isolates. Scale bar indicates genetic distance. Branch lengths represent pairwise Hamming distances (uncorrected *p* distances).

The decrease in LD (*R*^2^) with physical distance for the two major *L. rhizohalophila* subpopulations is shown in Fig. 2A. We observed higher LD values in group 2 compared with group 1. LD decay was relatively slower for group 2 with an LD50 of 520 bp, while LD decayed rapidly and reached half its maximum value (LD50) at ~120 bp in group 1. This contrasting LD decay pattern suggests that the frequency of genetic recombination also differed between the two groups.

Organization of the mating-type (*MAT*) loci and its flanking regions revealed that all individuals in group 1 and group 2 carry both *mat1-2-1* and *mat1-1-1* idiomorphs in a homothallic gene arrangement (Fig. 2B). We further assessed their evolutionary relationships while considering the possible occurrence of a recombination signal within or between groups. Group 1 was characterized by a complex network with many reticulations; by contrast, a star-shaped pattern was recovered in group 2 (Fig. 2C). Thus, this recombination pattern suggests that group 1 is more likely to be panmictic, while group 2 is more likely to be clonal. The disparity in nucleotide diversity between the two groups supported the conclusion. The genetic diversity of group 1, estimated by the average pairwise divergence (*θπ* = 0.0028) and the proportion of polymorphic sites per base (*θw* = 0.0025), was much higher than that in group 2 (*θπ* = 0.0009 and *θw* = 0.0010) (Wilcoxon’s test with *p* value < 2.2 × 10^−16^) (Table 1).

**Table 1:** Population genomics parameters of 29 *L. rhizohalophila* isolates.

Group	*θπ*	*θw*		Tajima’s D
All	0.0021		0.0028	−0.75
Group 1	0.0028		0.0025	0.54
Group 2	0.0009		0.0010	−0.32

### Genome-wide divergence of SNP and CNV profiles

We then assessed the genetic differences between the two subpopulations, searching for loci under adaptive selection. A total of 696,045 SNPs were called from all individuals from *L. rhizohalophila* groups 1 and 2. We found that differentiation between the subpopulations was high with an average Fst = 0.563 (95% CI 0.562–0.565) and the mean divergence Dxy = 0.27% (95% CI 0.26–0.27%). Unexpectedly, only approximately 5.3% of the SNPs were shared between the two groups and 11.9% (83,136) of the sites were reciprocally fixed for different alleles in the two groups. The remaining 82.8% of the SNPs were variable in one group and fixed in the other (Table 2). This distribution of shared polymorphism and fixed differences, together with high levels of divergence, may suggest an ancient split between the two groups.

**Table 2:** Number and percentage of polymorphic and fixed SNPs between group 1 and group 2 (total number of SNPs = 696,045).

	SNPs	%
Polymorphic in both group	36,670	5.3
Fixed in both group	83,136	11.9
Fixed in group 1	172,914	24.8
Fixed in group 2	403,325	58.0

**Fig. 3:**
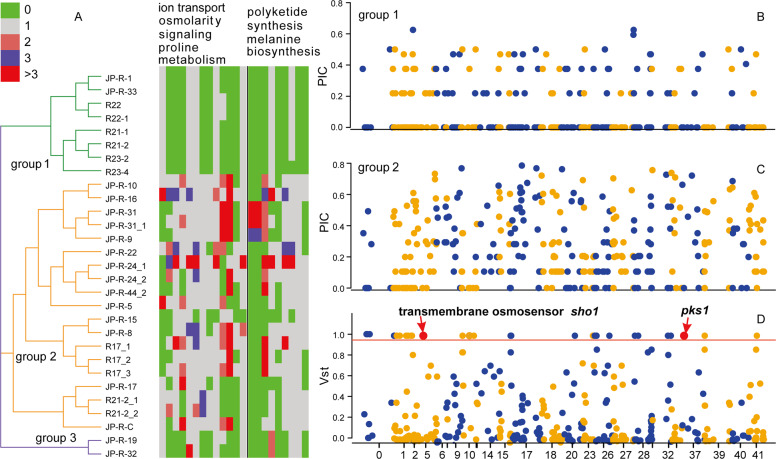
Genome-wide copy number variation profiles across the 29 *L. rhizohalophila* individuals. Loci encompassing salinity-responsive and melanin-biosynthetic genes with highly differentiated copy numbers between *L. rhizohalophila* groups are outlined. **A** Loci encompassing salinity-responsive and melanin-biosynthetic genes with highly differentiated copy numbers between *L. rhizohalophila* groups are outlined. **B**, **C**, **D** PIC and Vst values (y-axis) calculated with overlapping 10-kbp windows across all the contigs (x-axis) are plotted. The horizontal red line represents Vst values of 1.

We also assessed the pattern, selection, and diversity of CNVs using a sequence read-depth approach (Fig. S2). We found a total of 317 CNVs (139 duplications and 218 deletions) (Table S3 and Fig. S3). In total, 13.97 Mbp (22.7%) of the *L. rhizohalophila* genome contains CNVs, whereas only 1.24 Mbp (2%) is affected by SNPs. We detected a total of 1279 genes with significant CNV differences (Welch’s two-sample *t*-test, false discovery rate corrected *p-*value < 0.01) between the two groups, including 718 genes with putative functions and 561 genes with unknown functions. We observed a striking decrease in gene copies in group 1 and an expansion of gene duplications in group 2 (Fig. 3A). We further characterized patterns of CNV within populations by independently calculating the Polymorphic Information Content (PIC). The average PIC values were 0.095 and 0.242 for groups 1 and 2, respectively, reflecting the lower level of CNV in group 1 (Fig. 3B, C). Overall, our results suggest a faster turnover of copy numbers for group 1 compared with group 2, which can be explained by more frequent recombination in group 1 (Fig. 2C).

Estimates of Vst for CNVs revealed a number of highly differentiated loci that may be indicative of group-specific selective pressures; the average Vst was 0.255 (95% CI 0.219–0.290) (Fig. 3D). We found that a total of 36 CNVs overlapped 117 genes with extreme Vst values of 1.0. Although no GO term was enriched, small subsets of genes were functionally related to salinity adaptation (ion channels, melanin biosynthesis, etc.). For example, the polyketide synthase (*pks1*) and the transmembrane osmosensor are thought to be putatively involved in melanin biosynthesis and the high osmolarity-glycerol signaling pathway, respectively.

### Footprints of positive selection in the genome

**Fig. 4:**
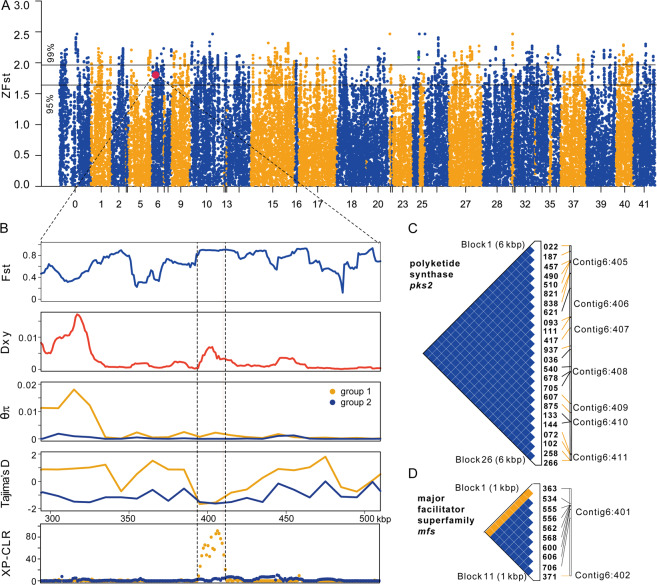
Genetic differentiation and detection of positive selection signals in the *L*. *rhizohalophila* population. **A** Genome-wide distribution of the Z-transformed fixation index ZFst plotted along contigs between group 1 and group 2. Scaffolds are arranged on the *x*-axis according to the reference genome and are separated by color. Genes within every 10-kbp window are plotted in a single column, and the 95% and 99% outliers are marked. **B** Enlargement of a genomic island of divergence (containing six genes) in scaffold 6, in which a strong positive selection signal in group 1 is detected. The genomic region showing high Fst, low nucleotide diversity (*θπ*), negative Tajima’s D, and high CLR values in group 1 are highlighted. **C**, **D** LD analysis of the *pks2* and *mfs* genes plotted by Haploview v4.1. The number and rectangle above the LD plot represent the position of the corresponding exons. The color of the plot corresponds to the strength of LD between sites, with the blue color representing strong LD and the yellow color representing weak or no LD. The triangle with a black bold line denotes LD blocks.

We calculated Tajima’s D to identify deviation from the neutral model of molecular evolution. Tajima’s *D* values were negative across the entire *L. rhizohalophila* population (−0.74) and the entire genome of group 2 (−0.32), perhaps as a result of demographic processes (population expansion) affecting the entire genome equally. In contrast, Tajima’s D was slightly positive for group 1 (0.54) (Table 1). In total, we found 1176 genes with outlier ZFst values exceeding the 95th percentile (Fig. 4A). Of these, 173 and 123 genes in group 1 and group 2, had Tajima’s D values below −1.0, respectively. GO enrichment analysis revealed that cellular components including the categories “intrinsic component of membrane,” “integral component of membrane,” and “membrane part” were significantly enriched in group 1 specifically (uncorrected *p* value < 0.05, Fig. S4), suggesting that the salinity-responsive systems have experienced group-specific activation and potentially positive selection.

To further identify patterns of genomic variation indicative of positive selection, we calculated genome-wide distribution of Tajima’s D using both sliding window and nonparametric empirical permutation approaches [50], to confirm that these peaks of population differentiation represent foci of selection. Briefly, we defined regions of the genome experiencing putative positive selection when statistic values of a given locus fell below the fifth percentile of the genomic and null distribution (Fig. S5). These criteria resulted in a cut-off of Tajima’s D statistic values of ≤ −1.31 and −1.34 for group 1 and group 2, respectively. If this stringent criterion is used, most of the salinity-responsive genes maintained the signature of positive selection.

Of note, we identified a genomic region (~20 kbp in length) located on scaffold 6 that had a strong signal of positive selection in group 1 individuals. This genomic region appears to encode a ~47 kbp melanin biosynthetic gene cluster, containing six genes related to melanin metabolism (polyketide synthase *pks2*, protein ID 2362; melanocyte-stimulating hormone receptor *mshr*, protein ID 2359), transport (major facilitator superfamily transporter *mfs*, protein ID 2361), transcriptional regulation (transcription factors ID 2358 and 2363) and cell wall remodeling (expansin, protein ID 2360). This region had low levels of genetic variation compared to the genome-wide average and had extremely negative Tajima’s D values in both groups (Fig. 4B). This pattern is consistent with recent and strong positive selection, although low absolute nucleotide diversity might also stem from the reduced mutation rates in these regions. An XP-CLR test also revealed a high but narrow CLR peak at the sweeping site of this region under the 0.01 significance level in group 1 (Fig. 4B). More specifically, the genes *pks2* (XP-CLR = 85.4, top 1% cutoff = 15.09, Tajima’s *D* = −1.85) and *mshr* (XP-CLR = 39.7, Tajima’s *D* = −1.83) showed strong signatures of selective sweeps. Subsequently, pairwise LD was examined in this specific region using Haploview to determine the extent of the putative selective sweep. A strong pattern of LD surrounding the *pks2* and *mfs* genes was observed in group 1 but not in group 2 (Fig. 4C, D). Moreover, the *pks2* gene contained 10 non-synonymous SNPs fixed or nearly fixed in group 1 with a major allele frequency of 90%, while only three non-synonymous SNPs in group 2 (Fig. S6).

### Impact of salinity on melanin synthesis and mycelial growth

**Fig. 5:**
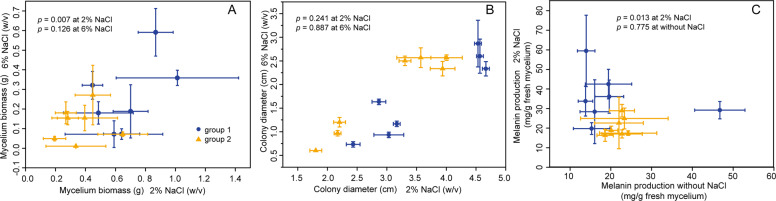
Melanin synthesis and mycelial growth of representative isolates from group 1 and group 2. For measurement of colony diameter and biomass, 2% and 6% NaCl levels were used. **A** Biomass comparison between the two groups under salinity stress conditions. **B** Growth rate on the PDA plates added with 2% and 6% NaCl. **C** Comparison of melanin production between the two groups under 2% NaCl and without NaCl. Data are averages from four biological replicates of the experiment. Error bars represent the standard deviation. Student’s *t*-tests were used to determine significant differences between the two groups. For each comparison, the *p* values are shown.

Owing to the high sequence divergence of the genomic island encoding a putative melanin biosynthetic gene cluster, we hypothesized that melanin production and growth under salinity stress may also differ dramatically between the two subpopulations. We thus measured the mycelial growth and melanin accumulation in seven representative isolates of each group across two salinity levels. We found that isolates of group 1 accumulated melanin to a lesser extent than group 2 isolates under the absence of NaCl, but the difference was not statistically significant. In contrast, average colony diameter and biomass accumulation were significantly higher in group 1 isolates compared with group 2 isolates (two-tailed *t*-test, *p* value = 0.007) in the 2% NaCl treatment (Fig. 5A, B). The melanin content of group 1 isolates was also significantly higher (two-tailed *t*-test, *p* value = 0.013) than that of group 2 isolates in the 2% NaCl medium (Fig. 5C), indicating that group 1 is more adapted to saline stress than group 2. The 6% NaCl treatment hampered fungal growth and measurements of melanin content cannot be completed.

## Discussion

Stress and evolution are always heavily intertwined [9]. In this work, we aimed to understand how the *L. rhizohalophila*–*S. salsa* symbiosis adapts to high saline soils by conducting population genomics analyses. We identified cryptic subpopulations of *L. rhizohalophila*, referred to as groups 1 and 2. We characterized a high level of genetic differentiation between these two lineages, suggesting a limited gene flow between subpopulations. Each group displayed a distinctive pattern of genetic recombination. Although the homothallic nature of group 2 individuals likely explains the observed low level of genetic recombination due to frequent inbreeding [57], it is still unclear why and how the extensive recombination is taking place in group 1. One possibility is that genetic exchange through parasexual recombination would occur and contribute to the observed genetic diversity [58].

The lack of physical barriers to migration and gene flow in our fine-scale study area suggests that the observed phenotypic differences are likely driven by microhabitat heterogeneity of saline soils imposing strong selection pressures. For instance, sea tides generate dramatic seasonal changes in salinity and other physicochemical factors in salt marsh soils [49]. Such environmental changes could potentially promote the evolution of stable gene polymorphisms. Allelic variants at specific loci would then reflect an ongoing process of adaptation to heterogeneous conditions [59, 60]. This has been suggested to explain the fine-scale genetic divergence of the teleost fish *Fundulus heteroclitus* populations, inhabiting three concurrent microhabitats within a single salt marsh [49]. In our study, we were not able to measure the physicochemical characteristics of the soils where *S. salsa* with its *L. rhizohalophila* endophytes was collected. This should be carried out in future studies to assess the ecological gradient taking place in this habitat.

**Fig. 6:**
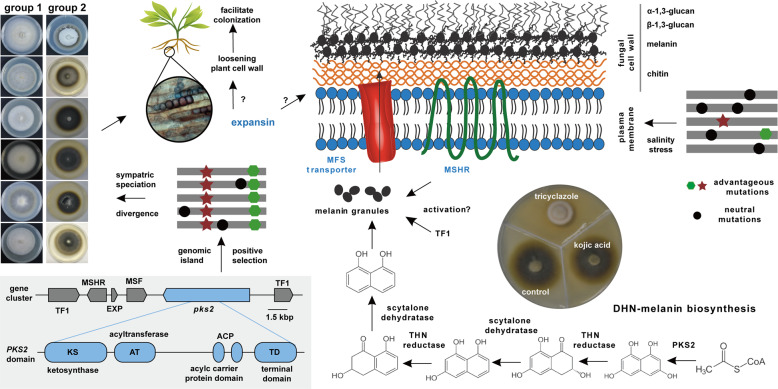
A schematic outline showing the potential effect of positive selection on the melanin biosynthetic gene cluster in *L. rhizohalophila*. PKS2 catalyzes the first step of melanin biosynthesis. The use of melanin biosynthesis inhibitors (tricyclazole and kojic acid, 50 μg mL^−1^) confirmed that *L. rhizohalophila* synthesized dihydroxynaphthalene melanin (DHN-melanin), as colonies growing in the presence of kojic acid, the inhibitor of dihydroxyphenyalanine (DOPA) melanin, appeared normal and were indistinguishable from the controls. The representative isolates from group 1 and group 2 grown on PDA without NaCl for two weeks at 25 °C are shown. Positive selection acting on this genomic island results in the fixation of many beneficial non-synonymous mutations. Using antiSMASH 5.0, the cluster organization (six genes) and PKS2 domains, including KS, AT, ACP, and TD were predicted *in silico*. The proposed model of melanin metabolism involving melanin biosynthesis (PKS2 and MSHR), membrane trafficking (MFS), regulation (two transcription factors), localization, and deposition (loosening of cell wall regulated by expansin) is shown. PKS polyketide synthase, MSHR melanocyte-stimulating hormone receptor, MFS major facilitator superfamily, TF transcription factors, EXP expansin, THN tetrahydroxynaphthalene.

Melanin biosynthesis is considered a major adaptive trait in fungi [7, 9]. Our results suggested that *L. rhizohalophila* is undergoing adaptation to salinity stress and this process appears to involve a ‘genomic island of divergence’ related to melanin biosynthesis. This gene arrangement may reduce the ability of recombination to break up favorable combinations of alleles as the frequency of recombination is greater in group 1 (Fig. 2) [61]. Of these genes, *pks2* is of particular interest, as most filamentous fungi synthesize melanin *via* a PKS pathway (Fig. 6) [62, 63]. We also identified a gene encoding a melanocyte-stimulating hormone receptor (MSHR) which is under positive selection. As an integral component of the plasma membrane, MSHR has been found only in mammals where it contributes to skin pigmentation [64]. However, there is a unified cellular principle for melanization in mammals and fungi [65]. We thus speculate that both *pks2* and *mshr* genes are involved in the increased melanin biosynthesis in *L. rhizohalophila*. In fungi, gene clusters for the synthesis of secondary metabolites generally encode transporter proteins, especially major facilitator superfamily (MFS) transporters [66]. Thus, the *mfs* gene found in the PKS cluster might be involved in the transport of melanin from the cytosol to the cell wall. The role of the expansin-like gene located in the PKS cluster remains unclear. Expansins act as loosening agents to increase plant cell growth and fungal expansins function in plant root colonization [67]. Given the fact that physically linked genes usually belong to the same metabolic network, it is tempting to speculate that the expansin-like protein loosens the fungal cell wall for increased melanin deposition. We propose that the genomic island contains a melanin biosynthetic gene cluster involved in melanin biosynthesis (PKS2 and MSHR), membrane trafficking (MFS), regulation (two transcription factors), and remodeling (loosening cell wall regulated by expansin) (Fig. 6).

Surprisingly, it seems that deletion of *pks1* does not reduce the melanin production in group 1 when challenged by salinity stress. The predicted coding sequences of *pks1 (protein ID 12252)* and *pks2* were 741 bp and 6339 bp in length, respectively. Interestingly, in silico analysis using antiSMASH 5.0 revealed that the *pks1* only had a ketosynthase (KS) domain but lacked the essential acyltransferase (AT) and acyl carrier protein (ACP) domains (Fig. 6), indicating that the function of *pks1* may be impaired.

Our data showed that group 1 isolates exhibited a growth advantage and higher melanin accumulation relative to group 2 isolates when incubated under 2% NaCl conditions. This suggests that increased melanization facilitates the adaptation to high-salinity environments, although it remains to assess how *L. rhizohalophila* hyphae inhabiting the host roots are affected by the external, high soil salinity.

## Conclusions

In this study, we uncovered the fine-scale population structure and ecological divergence of the endophyte *L. rhizohalophila* associated with the roots of the plant halophyte *S. salsa*. Both functional and genetic evidence support the role of a genomic island encoding a gene cluster, involved in biosynthesis, membrane trafficking, regulation, and localization of melanin, in shaping a melanization-associated phenotype (Fig. 6). Differences in mycelial growth yield and melanin biosynthesis between *L. rhizohalophila* subpopulations grown under saline stress conditions suggest that this genomic island contributes to the observed differences in melanin accumulation. Our findings provide a better understanding of the genetic and evolutionary mechanisms (e.g., melanin synthesis) underlying the adaptation to saline conditions of the *L. rhizohalophila–S. salsa* symbiosis. Population genomics studies of additional DSE symbioses will confirm whether melanization is an adaptation to extreme environments.

## Supplementary information

Supporting text information S1

Supplementary legends

Fig. S1

Fig. S2

Fig. S3

Fig. S4

Fig. S5

Fig. S6

Table S1

Table S2

Table S3
