# Correction: Divergence of a genomic island leads to the evolution of melanization in a halophyte root fungus

**DOI:** 10.1038/s41396-021-01083-w

**Published:** 2021-08-18

**Authors:** Zhilin Yuan, Irina S. Druzhinina, John G. Gibbons, Zhenhui Zhong, Yves Van de Peer, Russell J. Rodriguez, Zhongjian Liu, Xinyu Wang, Huanshen Wei, Qi Wu, Jieyu Wang, Guohui Shi, Feng Cai, Long Peng, Francis M. Martin

**Affiliations:** 1 State Key Laboratory of Tree Genetics and Breeding, Chinese Academy of Forestry, Beijing, China; 2 Research Institute of Subtropical Forestry, Chinese Academy of Forestry, Hangzhou, China; 3 Fungal Genomics Laboratory (FungiG), College of Resources and Environmental Sciences, Nanjing Agricultural University, Nanjing, China; 4 Department of Food Science, University of Massachusetts, Amherst, MA USA; 5 State Key Laboratory of Ecological Pest Control for Fujian and Taiwan Crops, College of Plant Protection, Fujian Agriculture and Forestry University, Fuzhou, China; 6 Department of Molecular, Cell and Developmental Biology, University of California, Los Angeles, CA USA; 7 Department of Plant Biotechnology and Bioinformatics, Ghent University, Ghent, Belgium; 8 VIB Center for Plant Systems Biology, Ghent, Belgium; 9 Centre for Microbial Ecology and Genomics, Department of Biochemistry, Genetics and Microbiology, University of Pretoria, Hatfield, South Africa; 10 Adaptive Symbiotic Technologies, University of Washington, Seattle, WA USA; 11 Key Laboratory of National Forestry and Grassland Administration for Orchid Conservation and Utilization at College of Landscape Architecture, Fujian Agriculture and Forestry University, Fuzhou, China; 12 State Key Laboratory of Mycology, Institute of Microbiology, Chinese Academy of Sciences, Beijing, China; 13 Key Laboratory of Plant Resources Conservation and Sustainable Utilization, South China Botanical Garden, Chinese Academy of Sciences, Guangzhou, China; 14 Beijing Advanced Innovation Center for Tree Breeding by Molecular Design, Beijing Forestry University, Beijing, China; 15 Université de Lorraine, INRAE, UMR Interactions Arbres/Micro-Organismes, Centre INRAE Grand Est Nancy, Champenoux, France

**Keywords:** Population dynamics, Fungal ecology, Population genetics

Correction to: *The ISME Journal* 10.1038/s41396-021-01023-8.

The original version of this article, unfortunately, contained a mistake in the affiliation of Prof. Yves Van de Peer:

^7^Department of Plant Biotechnology and Bioinformatics, Ghent University, Ghent, Belgium

^8^VIB Center for Plant Systems Biology, Ghent, Belgium

^9^Centre for Microbial Ecology and Genomics, Department of Biochemistry, Genetics and Microbiology, University of Pretoria, Hatfield, South Africa

The original article has been corrected.

