# Supplementary material for: Divergence of a genomic island leads to the evolution of melanization in a halophyte root fungus
Source: ISME J. 2021 Jun 9;15(12):3468–79. doi: 10.1038/s41396-021-01023-8 (PMC8629976; doi:10.1038/s41396-021-01023-8)
Supplement: Supplementary file 1 — Supporting text information S1 [file 41396_2021_1023_MOESM1_ESM.doc]

**Supporting information text S1**

**Methods**

**Genome sequencing**

Genomic DNA was extracted from a single spore culture using a modified cetyltrimethylammonium bromide (CTAB) protocol. Two sequencing libraries with insert sizes of 280 bp and 450 bp were constructed using NEBNext Ultra II DNA Library Prep Kit for Illumina (New England Biolabs, Massachusetts, USA). The libraries were then sequenced on an Illumina HiSeq X Ten System using a PE-150 module (KeGene Science & Technology Co. Ltd., China). In total, approximately 198 Gb of raw data were generated. The quality of all raw reads was assessed using FastQC [1]. The adaptors and low-quality bases were trimmed from the sequence using Trimmomatic with default parameters [2]. Finally, the process yielded approximately 12.8 Gb of clean reads for the *de novo* assembly of the JP-R-44 genome. The PacBio sequencing library was prepared using the SMRTbell template prep kit (Pacific Biosciences, Menlo Park, CA, USA). The constructed 20-kbp single-molecule real-time DNA sequencing library was sequenced on the PacBio Sequal platform and yielded over 6.49 Gb of subreads (mean length > 6 kbp and N50 length > 9 kbp).

**Reference genome assembly**

To estimate the genome size of JP-R-44, quality-filtered reads from the Illumina sequencing platform were subjected to the KmerGenie software (v1.7048) [3] with default parameter to estimate the best k-mer length for *de novo* genome assembly. The final result showed that the predicted best k-mer length was 81.

The genome of JP-R-44 was assembled by a hybrid strategy involving both PacBio and short read sequencing through the DBG2OLC pipeline available from https://github.com/yechengxi/DBG2OLC [4]. Firstly, the NGS contigs were preassembled using SparseAssembler [5] with some modifications (LD 0 GS 100000000 NodeCovTh 2 EdgeCovTh 1 k 81 g 15 BC 1). The generated contigs, together with Pacbio long reads, were used to overlap and layout using the DBG2OLC command with some modifications (LD 0 MinLen 500 k 17 KmerCovTh 2 MinOverlap 80 AdaptiveTh 0.01 RemoveChimera 1 ChimeraTh 2 ContigTh 2). Finally, the consensus scripts were run to concatenate the contigs and the raw reads for consensus. The preliminary assembly contigs were further corrected using Illumina reads by Pilon (v1.23, <https://github.com/broadinstitute/pilon>) [6] and scaffolds were assembled and optimized using Illumina short reads and PacBio long reads by OPERA (v2.0.6) [4, 7] with a k-mer size of 100. PBSuite (v15.8.24) was then used fill intra-scaffold gaps [8]. The completeness of this genome assembly was assessed using the BUSCO software (v4.0.1) [9, 10].

**Genome annotation**

For the detection of repetitive elements (TEs), RepeatMasker (v4.07) (http://repeatmasker.org/) was used against Repbase database (v23.06) (https://www.girinst.org/server/RepBase/) to identity known TEs. *De novo* TE annotation was performed using RepeatModeler (v1.0.11) (http://www.repeatmasker.org/RepeatModeler/) with default parameters.

Protein-coding genes were annotated based on homology prediction and *ab initio* prediction. For homology-based prediction, protein sequences of *Birnuria novae-zelandiae*, *Karstenula rhodostoma* and *Paraconiothyrium sporulosum* were obtained from a JGI Genome Portal at the MycoCosm database (https://genome.jgi.doe.gov/programs/fungi/index.jsf). The query protein sequences of these three species were subjected to against the repeat-masked JP-R-44 genome for homology proteins identification and gene structure prediction using Exonerate (v2.2.0) with modifications (--score 100 --percent 90) [11]. The ab initio prediction of protein-coding gene sequences was performed with Genemark-ES v4.10 using the masked genome [12]. The homology and *ab initio* based predicted genes were merged to form a comprehensive and non-redundant reference gene sets using EVidenceModeler (EVM, v1.1.1) [13]. The genes were functionally annotated by aligning them to the Non-redundant protein sequences (NR), eukaryotic orthologous groups of proteins (KOG), Kyoto Encyclopedia of Genes and Genomes (KEGG), Swissprot, and TrEMBL databases using RAPSearch2 (v2.22) [14] with HSSP criteria and also aligned to the Pfam database (<http://xfam.org/>) using hmmer (v. 3.2.1) [15] with parameter of e-value 1e-5. Gene Ontology (GO) terms were assigned to the genes using the BLAST2GO pipeline.

**References**

1. Andrews, S. FastQC: a quality control tool for high throughput sequence data. Available online at: <http://www.bioinformatics.babraham.ac.uk/projects/fastqc/>, 2010.
2. Bolger AM, Lohse M, Usadel B. Trimmomatic: a flexible trimmer for illumina sequence data. Bioinformatics. 2014;30:2114–2120.
3. Chikhi R, Medvedev P. Informed and automated k-mer size selection for genome assembly. Bioinformatics. 2014;30:31–37.
4. Ye C, Hill CM, Wu S, Ruan J, Ma ZS. DBG2OLC: Efficient Assembly of Large Genomes Using Long Erroneous Reads of the Third Generation Sequencing Technologies. Sci Rep. 2016;**6**:31900.
5. Ye C, Ma ZS, Cannon CH, Pop M, Yu D. W. Exploiting sparseness in *de novo* genome assembly. BMC Bioinformatics. 2012;13:S1.
6. Walker BJ, Abeel T, Shea T, Priest M, Abouelliel A, Sakthikumar S, *et al.* Pilon: an integrated tool for comprehensive microbial variant detection and genome assembly improvement. PLoS One. 2014; 9:e112963.
7. Gao S, Bertrand D, Chia BKH, Nagarajan N. OPERA-LG: efficient and exact scaffolding of large, repeat-rich eukaryotic genomes with performance guarantees. Genome Biol. 2016;17:102.
8. English AC, Richards S, Han Y, Wang M, Vee V, Qu J, et al. Mind the gap: upgrading genomes with Pacific Biosciences RS long-read sequencing technology. PLoS One. 2012;7:e47768.
9. Simão FA, Waterhouse RM, Ioannidis P, Kriventseva EV, Zdobnov EM. BUSCO: assessing genome assembly and annotation completeness with single-copy orthologs. Bioinformatics. 2015;31:3210–3212.
10. Waterhouse RM, Seppey M, Simão FA, Manni M, Ioannidis P, Klioutchnikov G, *et al.* BUSCO Applications from Quality Assessments to Gene Prediction and Phylogenomics. Mol Biol Evol. 2018;35:543–548.
11. Slater GSC, Birney E. Automated generation of heuristics for biological sequence comparison. BMC Bioinformatics. 2005;6:31.
12. Ter-Hovhannisyan V, Lomsadze A, Chernoff YO, Borodovsky M. Gene prediction in novel fungal genomes using an ab initio algorithm with unsupervised training. Genome Res. 2008;18:1979–1990.
13. Haas BJ, Salzberg SL, Zhu W, Pertea M, Allen JE, Orvis J, *et al.* Automated eukaryotic gene structure annotation using EVidenceModeler and the Program to Assemble Spliced Alignments. Genome Biol. 2008;9:R7.
14. Zhao Y, Tang H, Ye Y. RAPSearch2: a fast and memory-efficient protein similarity search tool for next-generation sequencing data. Bioinformatics. 2012;28:125–126.
15. Eddy SR. Accelerated Profile HMM Searches. PLoS Comput Biol. 2011;7:e1002195.
