# Supplementary material for: Divergence of a genomic island leads to the evolution of melanization in a halophyte root fungus
Source: ISME J. 2021 Jun 9;15(12):3468–79. doi: 10.1038/s41396-021-01023-8 (PMC8629976; doi:10.1038/s41396-021-01023-8)
Supplement: Supplementary file 2 — Supplementary legends [file 41396_2021_1023_MOESM2_ESM.doc]

**Fig. S1** Cross-validation error in the admixture analysis for K varying from 1–10 for the 29 *L. rhizohalophila* isolates.

**Fig. S2** Genome-wide copy number variation profiles across the 29 *L. rhizohalophila* isolates. Heatmaps depicting copy numbers are represented at the scaffold level, with different colors representing copy numbers of 0, 1, 2, 3, and *>* 3.

**Fig. S3** Distribution of CNV size in the *L. rhizohalophila* genome.

**Fig. S4** Gene Ontology (GO) enrichment analysis of 173 differentially expressed genes with Tajima’s D values less than -1.0 detected in group 1 of *L. rhizohalophila*. The top 15 enriched GO terms are shown.

**Fig. S5** Estimated genome-wide distribution plots for Tajima's D neutrality test statistics. Red lines represent the test statistic values calculated using the non-parametric permutation approach, and blue lines indicate the empirical sliding-window genome-wide distribution. The y-axis indicates the frequency of the test statistic value in the dataset.

**Fig. S6** The distribution of non-synonymous and synonymous SNPs of the strongly positively selected *pks2* gene between the two groups.
