# Supplementary figures and images for: Divergence of a genomic island leads to the evolution of melanization in a halophyte root fungus
Source: ISME J. 2021 Jun 9;15(12):3468–79. doi: 10.1038/s41396-021-01023-8 (PMC8629976; doi:10.1038/s41396-021-01023-8)

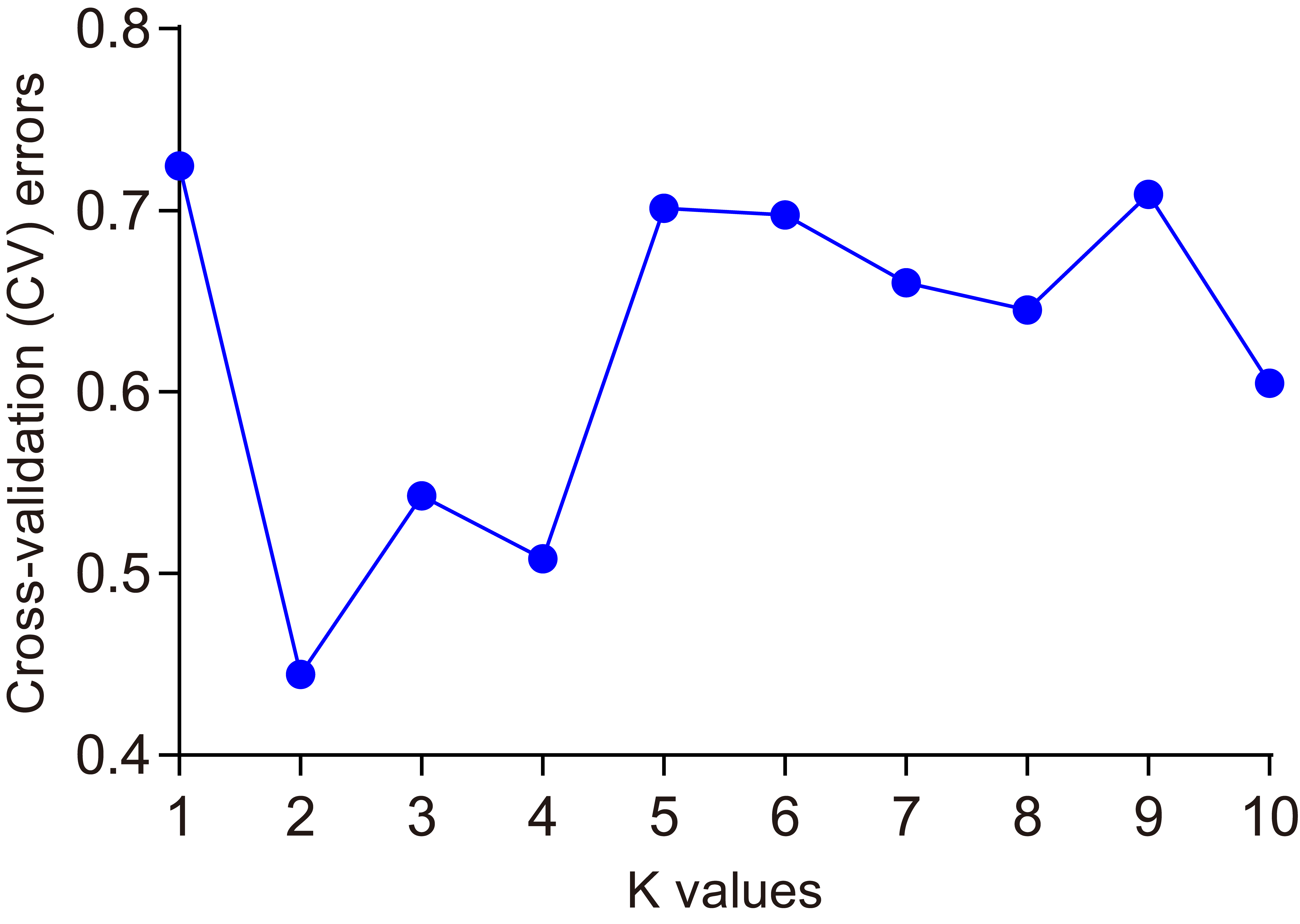

Supplement: Supplementary file 3 — Fig. S1 [file 41396_2021_1023_MOESM3_ESM.tif]

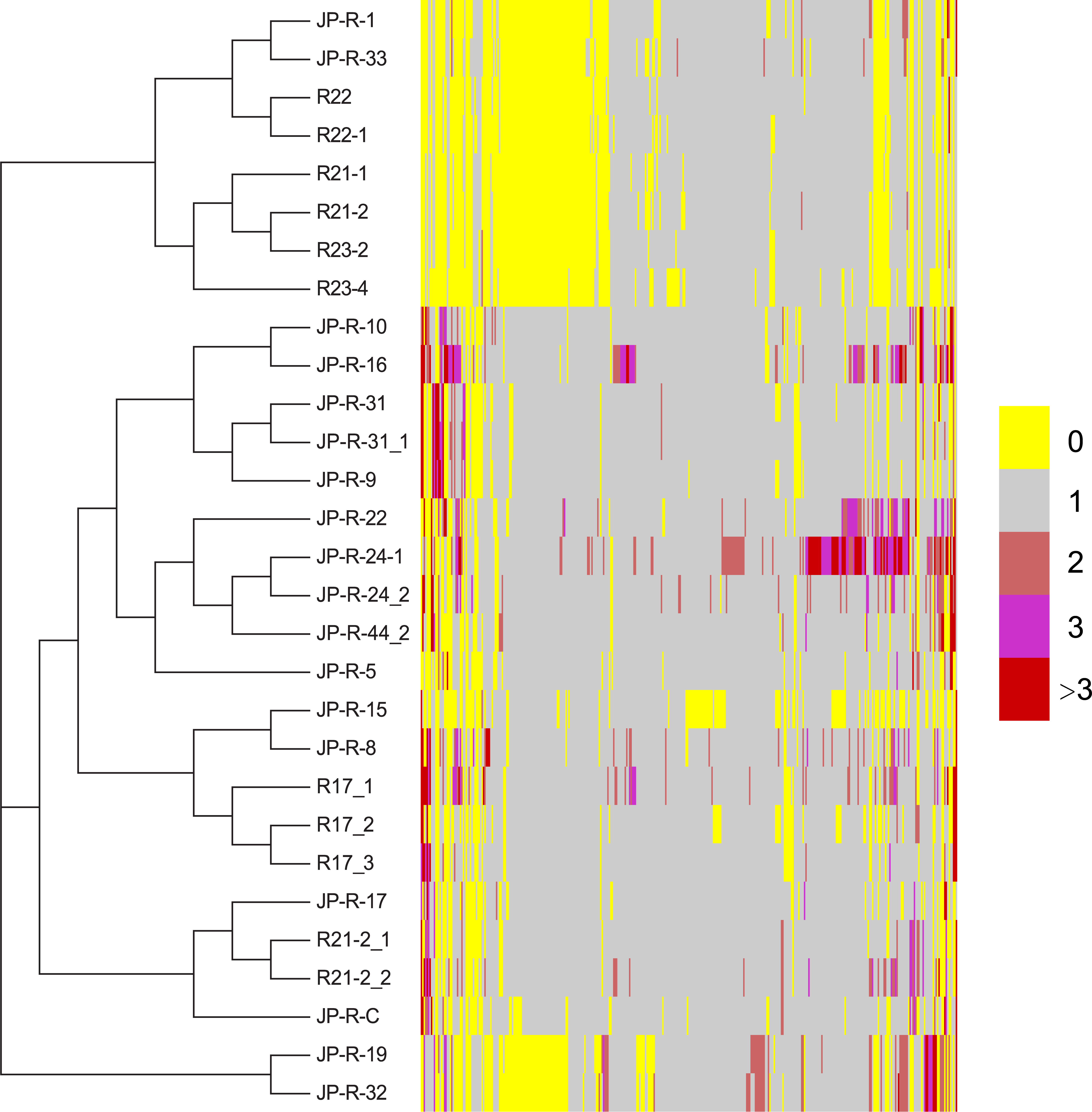

Supplement: Supplementary file 4 — Fig. S2 [file 41396_2021_1023_MOESM4_ESM.tif]

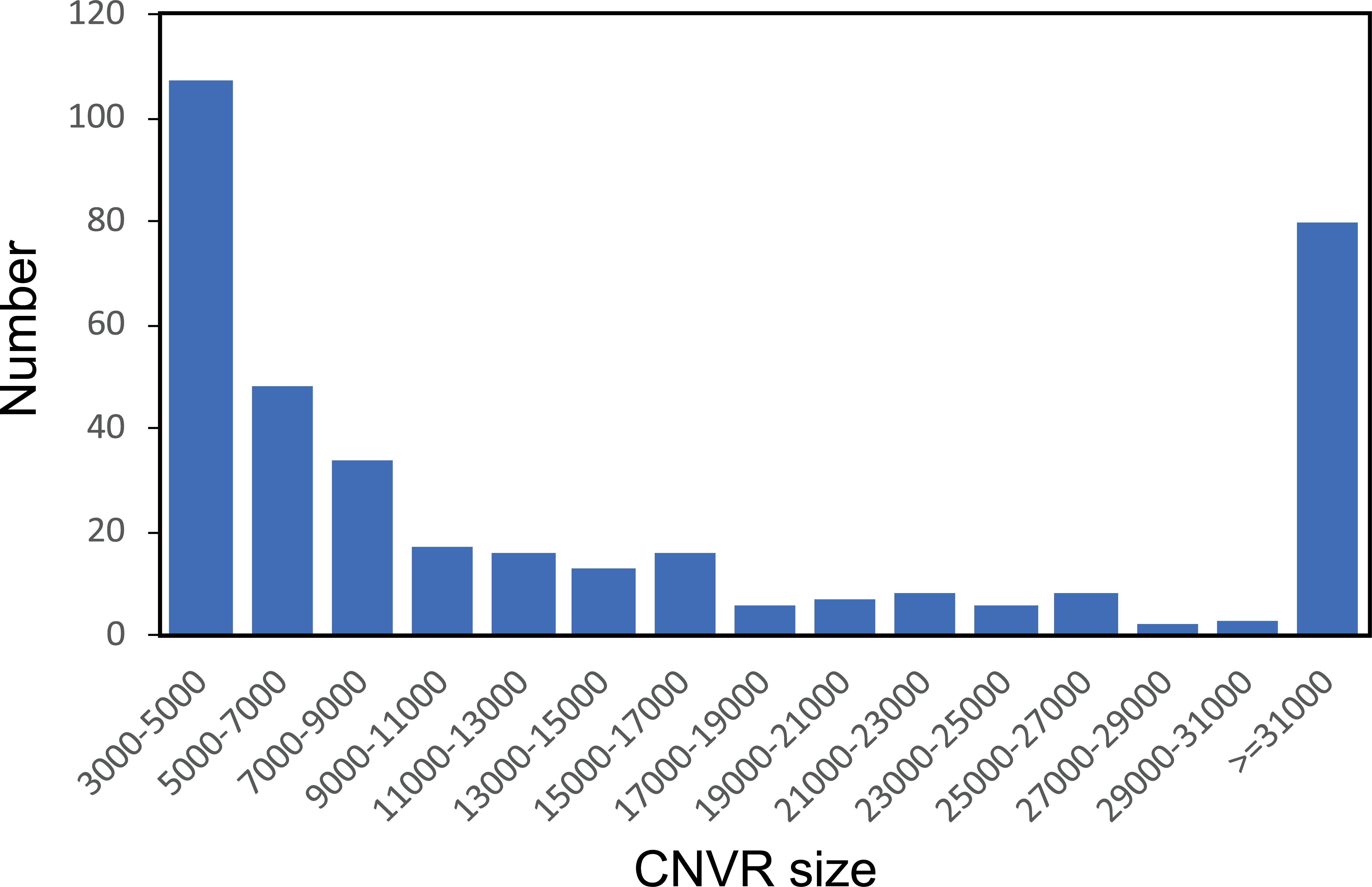

Supplement: Supplementary file 5 — Fig. S3 [file 41396_2021_1023_MOESM5_ESM.tif]

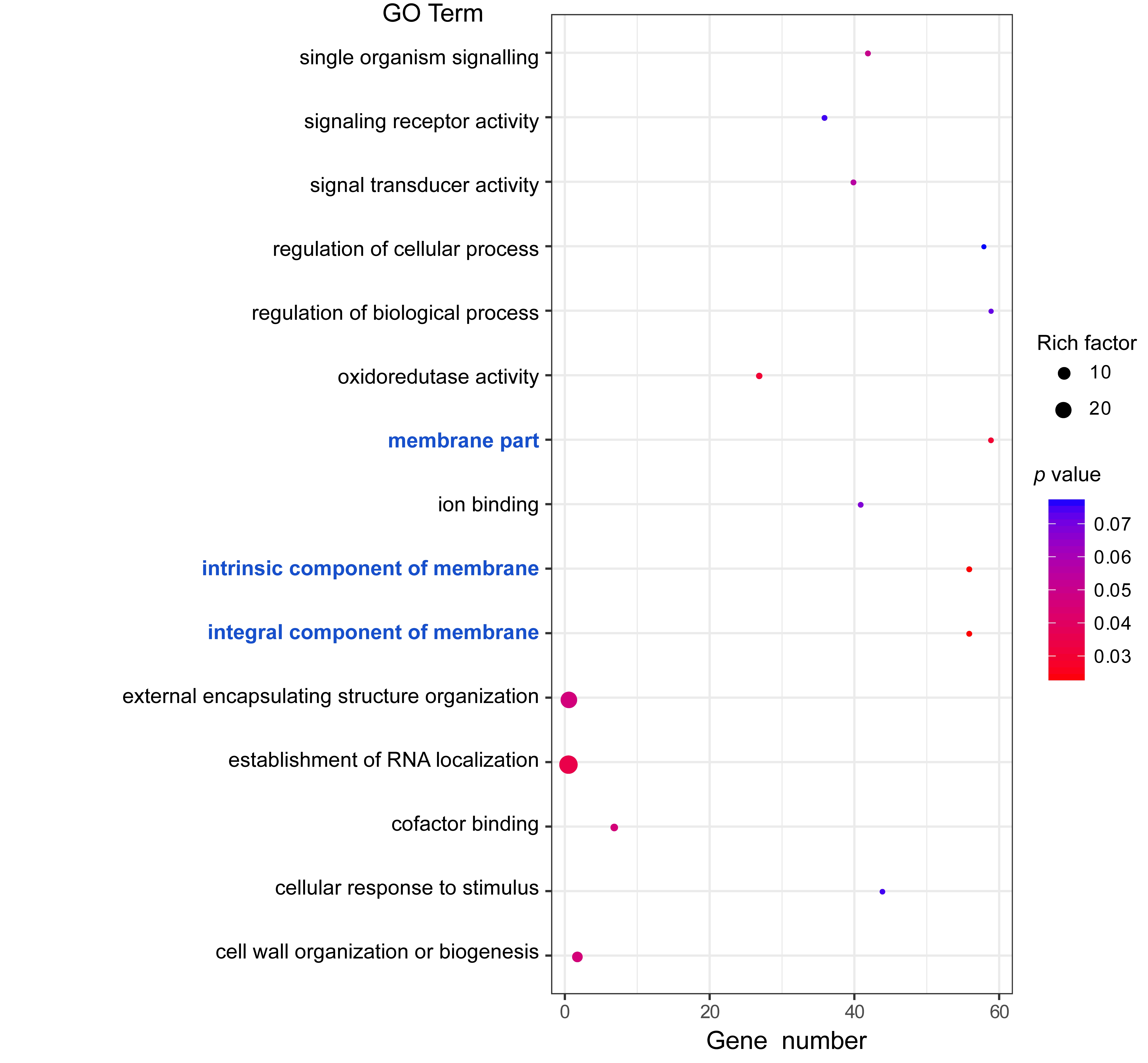

Supplement: Supplementary file 6 — Fig. S4 [file 41396_2021_1023_MOESM6_ESM.tif]

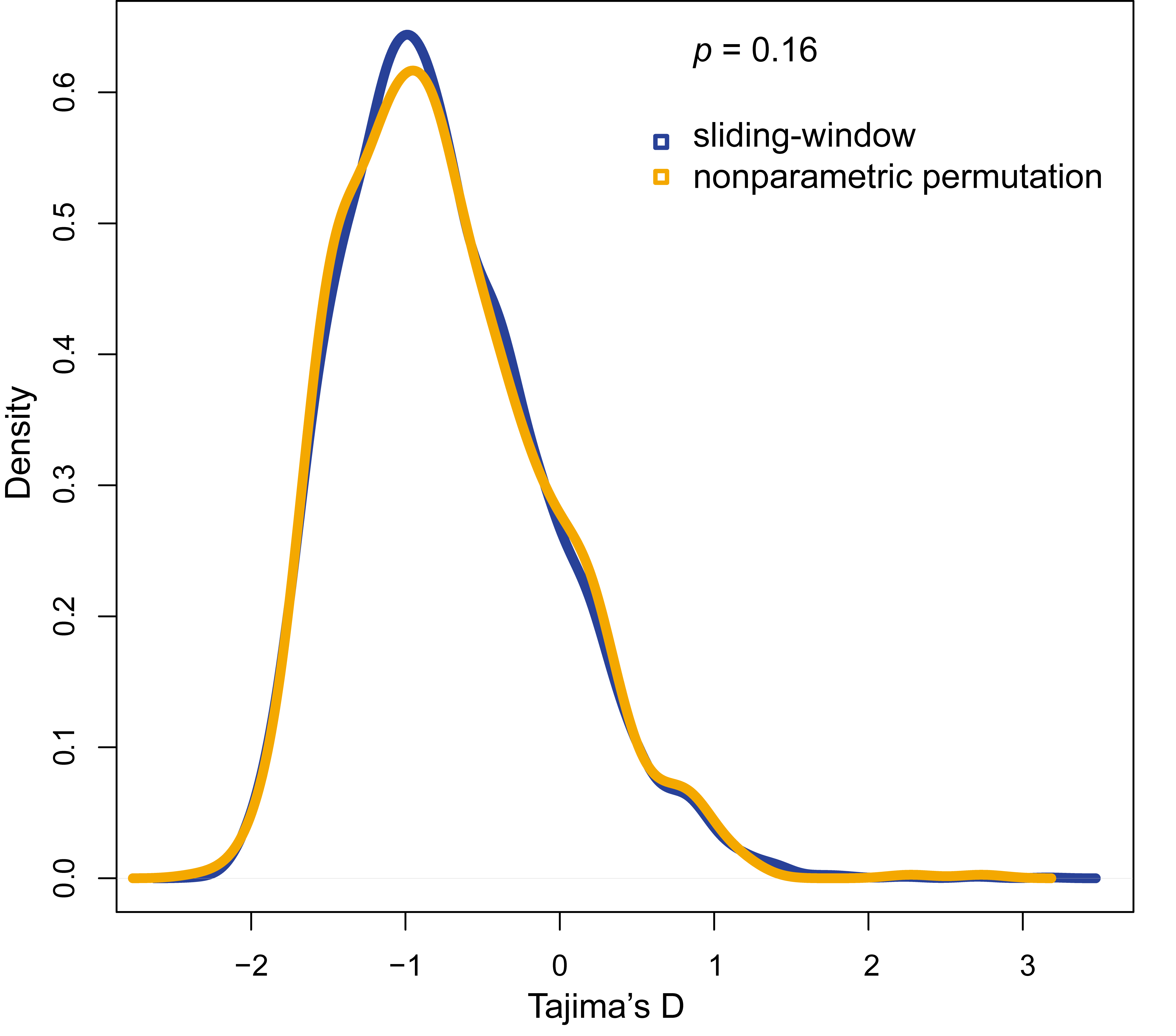

Supplement: Supplementary file 7 — Fig. S5 [file 41396_2021_1023_MOESM7_ESM.tif]

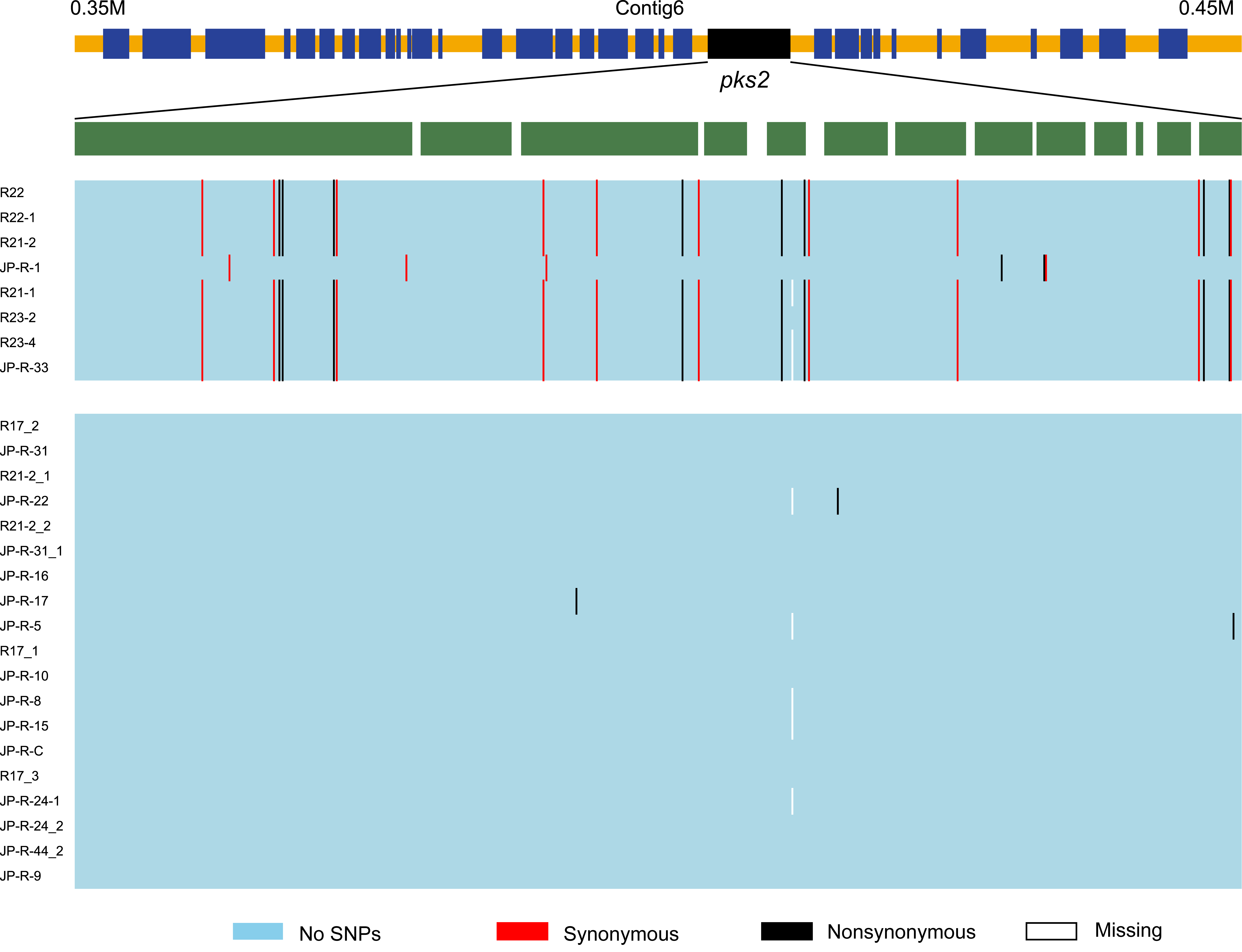

Supplement: Supplementary file 8 — Fig. S6 [file 41396_2021_1023_MOESM8_ESM.tif]
