# Supplementary material for: Divergence of a genomic island leads to the evolution of melanization in a halophyte root fungus
Source: ISME J. 2021 Jun 9;15(12):3468–79. doi: 10.1038/s41396-021-01023-8 (PMC8629976; doi:10.1038/s41396-021-01023-8)
Supplement: Supplementary file 9 — Table S1 [file 41396_2021_1023_MOESM9_ESM.doc]

**Table S1** Genome statistics for the *L*. *rhizohalophila* reference isolate

| Description | JP-R-44 |
| --- | --- |
| Genome assembly size (bp) | 61,456,043 |
| Number of contigs | 42 |
| N_50_ scaffold (bp) | 2,529,577 |
| N_90_ scaffold (bp) | 1,172,960 |
| Sequencing read coverage depth | 99.53 |
| Transposable elements (TEs) number | 68,309 |
| GC content (%) | 45.05 |
| Total number of genes | 14,646 |
| Average gene length (bp) | 1,644 |
| Average protein length (aa) | 434 |
| Average CDS length | 1,303 |
