# Supplementary material for: Divergence of a genomic island leads to the evolution of melanization in a halophyte root fungus
Source: ISME J. 2021 Jun 9;15(12):3468–79. doi: 10.1038/s41396-021-01023-8 (PMC8629976; doi:10.1038/s41396-021-01023-8)
Supplement: Supplementary file 11 — Table S3 [file 41396_2021_1023_MOESM11_ESM.doc]

**Table S3** CNV statistics in the *L. rhizohalophila* population.

| Total CNV number | 371 |
| --- | --- |
| Total CNV length (bp) | 13,967,958 |
| Average length of CNV (bp) | 37,649.5 |
| Number of CNV containing genes | 197 |
| Number of CNV-overlapped genes | 2,650 |
